# Ascorbic Acid for Prevention of Intraoperative Blood Loss and Related Complications During Myomectomy: A Systematic Review and Meta-Analysis of Randomized Controlled Trials

**DOI:** 10.7759/cureus.31571

**Published:** 2022-11-16

**Authors:** Ahmed Abu-Zaid, Hanaa Alrashidi, Arwa Almouh, Zainab M Abualsaud, Ahmed M Saleh, Sultan B Aldawsari, Mohannad M Alajmi, Osama Alomar

**Affiliations:** 1 College of Graduate Health Sciences, The University of Tennessee Health Science Center, Memphis, USA; 2 Department of Internship, Kuwait Institute for Medical Specializations, Kuwait City, KWT; 3 College of Medicine, Alfaisal Univeristy, Riyadh, SAU; 4 College of Medicine, Almaarefa University, Riyadh, SAU; 5 College of Medicine and Medical Science, Arabian Gulf University, Manama, BHR; 6 College of Medicine, The University of Jordan, Amman, JOR; 7 Department of Obstetrics and Gynecology, King Faisal Specialist Hospital and Research Centre, Riyadh, SAU

**Keywords:** meta-analysis, systematic review, hemorrhage, myomectomy, vitamin c, ascorbic acid

## Abstract

Leiomyomas are inherently well-vascularized neoplasms; thus, they are very vulnerable to bleeding-associated complications during myomectomy. Ascorbic acid has well-established functions in tissue healing and the prevention of bleeding tendencies. Several randomized controlled trials (RCTs) have explored the antihemorrhagic utility of ascorbic acid administration during myomectomy. This research aimed to systematically and meta-analytically summarize the clinical antihemorrhagic efficacy of ascorbic acid (i.e., the intervention arm) versus placebo/no treatment (i.e., the control arm) during myomectomy. We electronically searched six sources, i.e., PubMed, Scopus, Web of Science, Cochrane Central Register of Controlled Trials, and Google Scholar. Our search was from inception until October 2022. We used the Cochrane Risk of Bias Scale (version 2) to assess the quality of the included studies. We summarized the effect sizes as the mean difference (MD) or risk ratio (RR) with a 95% confidence interval (CI) in a fixed-effects or random-effects model. Overall, three RCTs met the inclusion criteria, comprising a total of 193 patients: 99 patients were allocated to the ascorbic acid arm, whereas 94 patients were allocated to the control arm. The overall study quality was "low" and "some concerns" risk of bias in two and one RCT(s), respectively. There was no significant difference between the ascorbic acid and control arms regarding the mean intraoperative blood loss (n=2 RCTs, MD = −190.29 ml, 95% CI [−626.62, 246.05], p=0.39) and mean change in hemoglobin level (n=3 RCTs, MD = −0.26 mg/dl, 95% CI [−0.56, 0.04], p=0.09), respectively. Conversely, the ascorbic acid arm had statistically significant reductions in the mean operative time (n=3 RCTs, MD = −24.10 min, 95% CI [−30.67, −17.53], p<0.001) and the rate of blood transfusion (n=3 RCTs, RR=0.36, 95% CI [0.15, 0.87], p=0.02) compared with the control arm. No serious adverse events related to ascorbic acid were identified. In conclusion, ascorbic acid administration was associated with several beneficial effects, including reductions in mean operative time and rate of blood transfusion, but without affecting the mean intraoperative blood loss and mean change in hemoglobin level. In view of the limitations of the present meta-analysis, the use of ascorbic acid as an antihemorrhagic additive among patients undergoing myomectomy is not strongly recommended.

## Introduction and background

Leiomyomas are non-cancerous neoplasms that originate from uterine smooth muscle cells. They are one of the most common neoplasms of the female genital system globally. Troublesome symptoms are present in roughly 33% of patients and comprise pelvic pain, excessive menstrual bleeding, and pressure symptoms. Iron deficiency anemia, preterm labor, and infertility are other serious long-term complications [1].

Medical therapy is the first-line treatment for patients with symptomatic leiomyoma. Nevertheless, when medical therapy fails, surgical intervention becomes necessary and comprises a myomectomy or hysterectomy. Notably, for patients desiring to preserve childbearing, myomectomy is the appropriate surgical intervention [2].

Leiomyomas are inherently well-vascularized neoplasms [3]; thus, they are very vulnerable to bleeding-associated complications during myomectomy [4]. Intraoperative hemorrhage is frequent [4], and it may necessitate blood transfusion [5]. Unbalanced hemostasis, circulatory collapse, and death are rare but serious bleeding-associated complications during myomectomy [6]. Overall, interventions to mitigate the magnitude of bleeding and related complications during myomectomy are considerably important.

Various mechanical (e.g., uterine artery ligation and uterine tourniquet application) and medical (e.g., vasopressin and tranexamic acid administration) methods have been investigated in the literature to lessen blood loss and related complications during myomectomy [4]. Nonetheless, most of these methods are susceptible to a dearth of adequate evidence to support their effectiveness [4].

Ascorbic acid (also known as vitamin C) has well-established functions in tissue healing and the prevention of bleeding tendencies [7,8]. Several randomized controlled trials (RCTs) have explored the antihemorrhagic utility of ascorbic acid administration during myomectomy [9-11]. The findings have been contradictory and limited by the small sample size. Additionally, no meta-analysis has been previously conducted. This research aims to systematically and meta-analytically summarize the clinical efficacy of ascorbic acid (i.e., the intervention arm) versus placebo/no treatment (i.e., the control arm) during myomectomy.

## Review

Methods

Research Protocol

We performed this study according to the guidelines provided by the Cochrane Handbook of Systematic Reviews of Interventions [12] and the Preferred Reporting Items for Systematic Reviews and Meta-Analyses (PRISMA) [13]. Ethical approval was not required due to the lack of individually identifiable data. The research protocol was not recorded in the International Prospective Register of Systematic Reviews (PROSPERO).

Inclusion and Exclusion Criteria

Our inclusion criteria involved: (i) studies with an RCT design; (ii) patients undergoing myomectomy; (iii) ascorbic acid was used as the intervention arm; (iv) placebo/no treatment was used as the control arm; and (v) studies reporting at least one of our efficacy endpoints, namely operative time (min), intraoperative blood loss (ml), mean change in hemoglobin level (mg/dl), and the rate of blood transfusion (%). On the other hand, our exclusion criteria involved: (i) non-RCT study designs, including observational studies, reviews, conference abstracts, and case reports; (ii) patients undergoing procedures other than myomectomy, such as hysterectomy; and (iii) interventions other than ascorbic acid.

Data Sources, Search Strategy, and Study Selection 

We electronically searched six sources, i.e., PubMed, Scopus, Web of Science, Cochrane Central Register of Controlled Trials, and Google Scholar. Our search was from inception until October 2022. We used the following search terms: (myomectomy OR fibroidectomy OR "uterine myomectomy" OR "uterine fibroidectomy") AND ("vitamin C" OR "ascorbic acid" OR ascorbate OR magnorbin OR hybrin). Independently, two coauthors completed the search for data sources, screened titles/abstracts, read the full text of potential citations, and finalized the list of eligible studies. Disputes were settled via discussion with the first author.

Quality Assessment of the Included Studies

We used the Cochrane Risk of Bias (ROB) scale (version 2) [14] to assess the quality of the included studies. This tool examines various domains, including the randomization process, deviation from the intended interventions, missing outcome data, outcome measurement, and selection of the reported result. We scored each domain as well as the overall quality as "low," "some concerns," or "high" risk of bias. Independently, two coauthors completed the quality assessment, and disputes were settled via discussion with the first author.

Data Outcomes

We collected information about the baseline characteristics of the included studies, such as the first author, year of publication, country, total sample size, study arms, type of myomectomy, and type of anesthesia. Besides, we collected information about the baseline characteristics of the included patients, such as sample size, dose, age, body mass index, number of myomas, and preoperative hemoglobin level. Lastly, we collected information about the efficacy endpoints, including operative time (min), intraoperative blood loss (ml), the mean change in hemoglobin level (mg/dl), and the rate of blood transfusion (%). Independently, two coauthors completed the data collection, and disputes were settled via discussion with the first author.

Meta-analysis

We used the Cochrane Collaboration's Review Manager program (version 5.4 for Windows) for meta-analysis. We analyzed the continuous data using the inverse variance method and summarized the overall effect size as the mean difference (MD) with a 95% confidence interval (CI). On the other hand, we analyzed the dichotomous data using the Mantel-Haenszel method and summarized the overall effect size as a risk ratio (RR) with a 95% CI. We determined between-study heterogeneity according to the I-square (I2) static and p-value of the chi-square test. Significant between-study heterogeneity was recognized when I2 measured >50% and the p-value of the chi-square test measured <0.1. We used the fixed-effects and random-effects models for statistical analysis of homogeneous and heterogeneous outcomes, respectively. For all endpoints, we determined statistical significance when the p-value measured ≤0.05.

Results

Summary of the Literature Search

**Figure 1:**
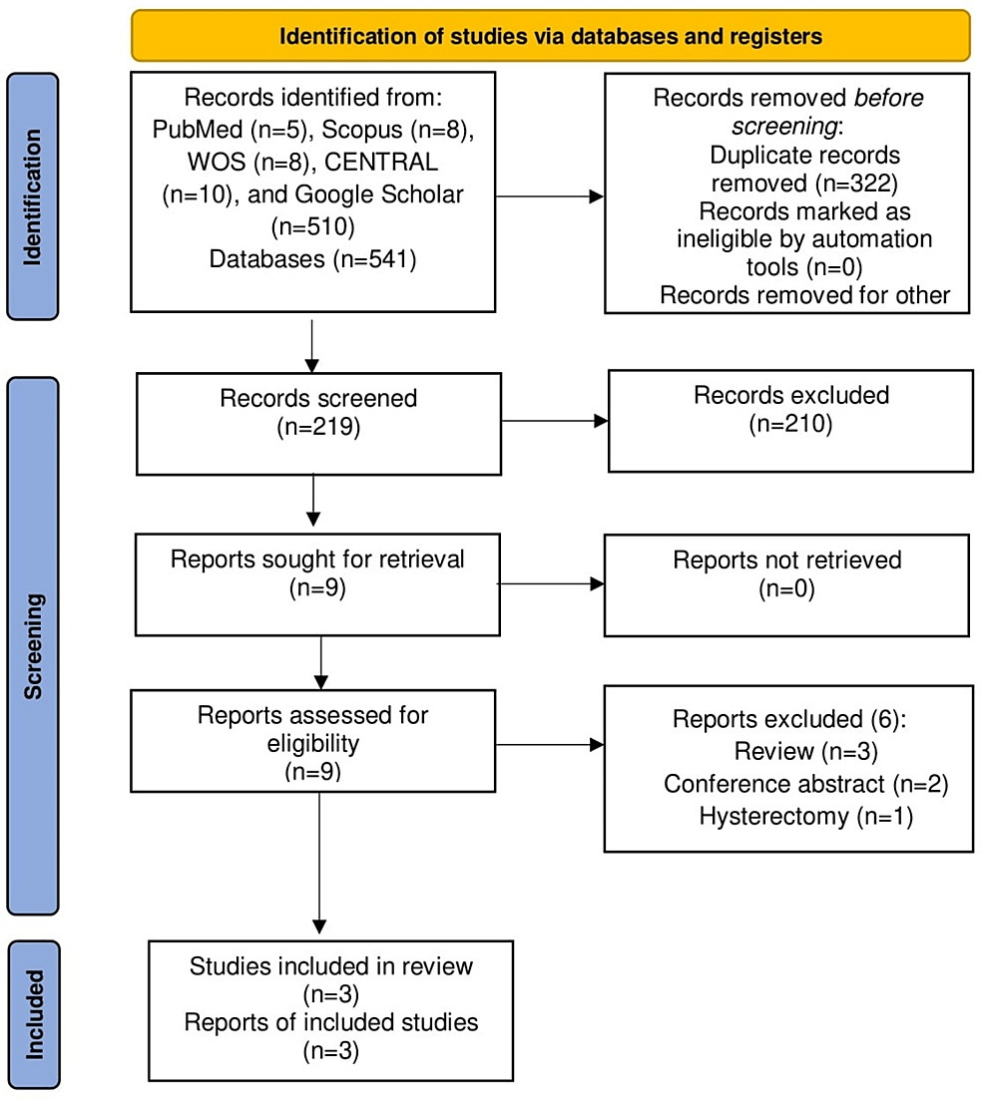
The Preferred Reporting Items for Systematic Reviews and Meta-Analyses (PRISMA) flowchart for literature search and study selection.

Figure 1 illustrates the PRISMA flowchart for literature search and study selection. The data search yielded 541 citations, 322 of which were discarded due to duplication. Of the 219 remaining citations, 210 were further discarded after the screening of titles/abstracts for various reasons, including irrelevance to the topic of research and meeting the exclusion criteria. Nine citations underwent full-text screening, of which six were excluded owing to various reasons, including review articles (n=3), conference abstracts (n=2), and patients undergoing hysterectomy (n=1). Eventually, three RCTs were included in the final analysis [9-11].

Summary of the Included RCTs and Participants

Tables 1-2 display a summary of the baseline characteristics of the included RCTs and participants, respectively. The three RCTs comprised a total of 193 patients; 99 patients were allocated to the ascorbic acid arm, whereas 94 patients were allocated to the control arm [9-11]. Two RCTs were conducted in South Korea [10,11] and one RCT was conducted in Iran [9]. Two RCTs performed myomectomy via laparoscopy [10,11] and one RCT performed myomectomy via laparotomy [9]. The dose of ascorbic acid was 2000 mg in two RCTs [10,11] and 500 mg in one RCT [9]. There was no significant difference between both arms regarding age, body mass index, number of leiomyomas, and baseline preoperative hemoglobin level.

**Table 1:** Summary of the baseline characteristics of the included studies.

Study identifier [reference]	Country	Total sample size (n)	Study arms		Type of myomectomy	Type of anesthesia
			Intervention	Control		
Hwang and Kim [11]	South Korea	45	Ascorbic acid	Placebo	Laparoscopic	General anesthesia
Lee et al. [10]	South Korea	46	Ascorbic acid	Placebo	Laparoscopic	General anesthesia
Pourmatroud et al. [9]	Iran	102	Ascorbic acid	Nothing	Abdominal	General anesthesia

**Table 2:** Summary of the baseline characteristics of the included participants. Age, body mass index, number of leiomyomas, and preoperative hemoglobin level were reported as means ± standard deviations.

Study identifier [reference]	Arm	Study arm	Sample size (n)	Dose (route)	Age (years)	Body mass index (kg/m²)	Number of leiomyomas	Preoperative hemoglobin (mg/dl)	
									Hwang and Kim [11]	Intervention	Ascorbic acid	23	2000 mg (intravenous)	42.3 ± 6.0	23.1 ± 3.1	1.3 ± 0.5	12.5 ± 1.61	
Control	Placebo	22	500 ml normal saline (intravenous)	40.3 ± 6.2	22.5 ± 2.49	1.5 ± 0.8	11.7 ± 1.94		
Lee et al. [10]	Intervention	Ascorbic acid	24	2000 mg (intravenous)	42 ± 6.38	23.1 ± 3.12	1.5 ± 0.93	12.6 ± 1.58	
	Control	Placebo	22	500 ml normal saline (intravenous)	42 ± 6.38	22.6 ± 2.50	1.6 ± 0.85	11.8 ± 1.94	
Pourmatroud et al. [9]	Intervention	Ascorbic acid	52	500 mg (intravenous)	32 ± 4.5	22.6 ± 2.50	1.96 ± 1.04	10.12 ± 0.9	
	Control	Nothing	50	Nothing	30.4 ± 6.5	22.6 ± 2.50	2.36 ± 1.56	10.05 ± 1	

Summary of the Quality Assessment

**Figure 2:**
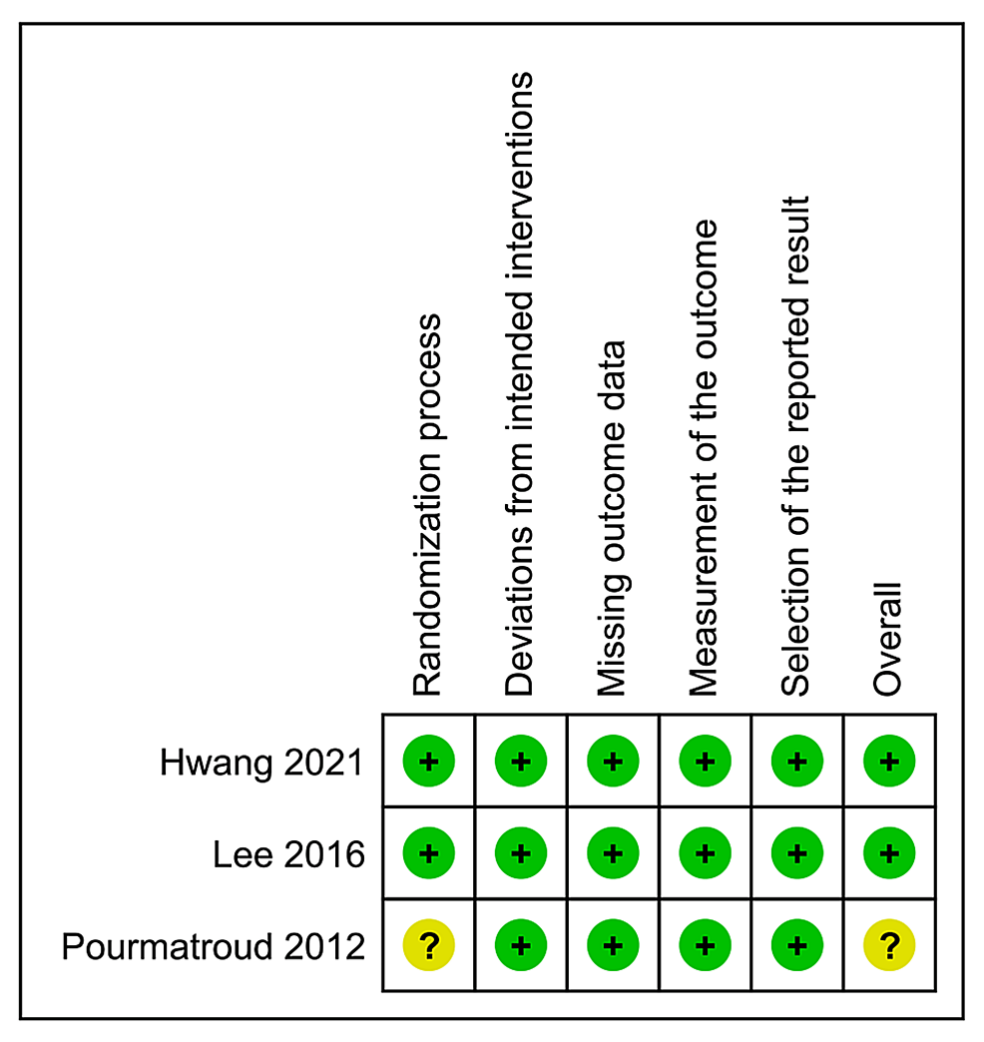
Summary of the quality assessment of the included studies. ?: some concerns of bias, +: low risk of bias, the cited articles [9-11].

Figure 2 illustrates the risk of bias summary of the included RCTs. Two RCTs [10,11] were evaluated as "low" risk of bias. However, one RCT [9] was evaluated as "some concerns" because it did not provide adequate details on the randomization process and allocation concealment.

Summary of the Efficacy Endpoints

**Figure 3:**
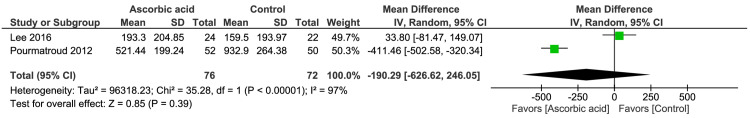
Meta-analysis of the intraoperative blood loss (ml) between the ascorbic acid and control arms. The cited articles [9,10].

**Figure 4:**
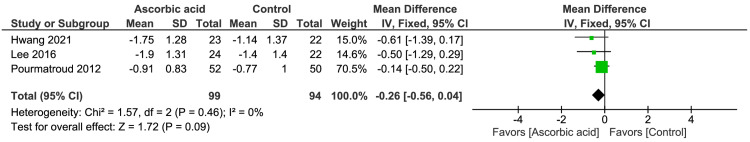
Meta-analysis of the mean change in hemoglobin level (mg/dl) between the ascorbic acid and control arms. The cited articles: [9-11].

**Figure 5:**
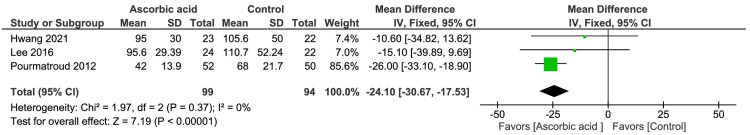
Meta-analysis of the mean operative time (min) between the ascorbic acid and control arms. The cited articles [9-11].

**Figure 6:**
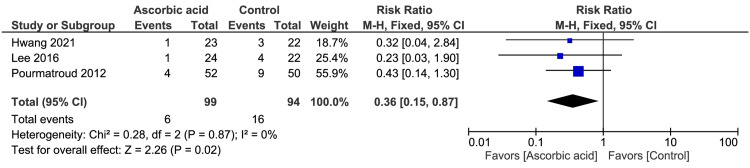
Meta-analysis of the rate of blood transfusion (%) between the ascorbic acid and control arms. The cited articles [9-11].

There was no significant difference between the ascorbic acid and control arms regarding the mean intraoperative blood loss (n=2 RCTs, MD = −190.29 ml, 95% CI [−626.62, 246.05], p=0.39, Figure 3) and mean change in hemoglobin level (n=3 RCTs, MD = −0.26 mg/dl, 95% CI [−0.56, 0.04], p=0.09; Figure 4), respectively. The pooled analyses were heterogeneous (chi-square p<0.001 and I2=97%) and homogeneous (chi-square p=0.46 and I2=0%), respectively. Conversely, the ascorbic acid arm had statistically significant reductions in the mean operative time (n=3 RCTs, MD = −24.10 min, 95% CI [−30.67, −17.53], p<0.001; Figure 5) and the rate of blood transfusion (n=3 RCTs, RR=0.36, 95% CI [0.15, 0.87], p=0.02; Figure 6), compared with the control arm. Both pooled analyses were homogeneous (chi-square p>0.1 and I2<50%).

Discussion

Summary of Main Results

This systematic review and meta-analysis examined the antihemorrhagic efficacy of ascorbic acid versus passive control (placebo/no treatment) among patients undergoing myomectomy. Overall, three RCTs met the inclusion criteria, comprising a sum of 193 patients. The overall study quality was "low" and "some concerns" risk of bias in two and one RCT(s), respectively. Overall, ascorbic acid administration was linked to significant reductions in mean operative time and rate of blood transfusion compared with the control arm. Nonetheless, there was no significant difference between both arms regarding the mean intraoperative blood loss and mean change in hemoglobin level.

Interpretation of Results and Clinical Implications

The increased bleeding tendency of leiomyomas during myomectomy can be partially ascribed to their hypoxic microenvironment [15] and the consequent enriched vascularity [3]. Proper maintenance of a blood-free surgical field is an essential outcome. This is because a bloody surgical field is risky because it may be linked to potential serious repercussions, such as opaque surgical visualization, unintentional injury to structures, and prolonged tissue repair [16].

Scurvy is a disease that results from ascorbic acid insufficiency. It is characterized by compromised collagen production and friable blood vessels [10]. The observations of a prolonged coagulation period and delayed platelet aggregation in patients with scurvy have proposed a substantial role for ascorbic acid in blood clotting [17,18]. Exogenous supplementation of ascorbic acid has been depicted to rescue these bleeding-related complications [7,8]. Mechanistically, ascorbic acid exhibits its antihemorrhagic effects by reducing the levels of reactive oxygen radicals and strengthening fibrin-platelet stability [19]. Preoperative administration of ascorbic acid to reduce intraoperative blood loss has been successfully implicated in patients undergoing hysterectomy [20] and colonoscopic resection of polyps [21].

In the present meta-analysis, ascorbic acid did not appear to reduce the extent of intraoperative blood loss. This can be ascribed to various reasons, including the small number of meta-analyzed studies (n=2), different methods of estimating intraoperative blood loss, different types of myomectomy, and widely dissimilar amounts of intraoperative blood loss [9,10]. Likewise, there was no significant difference between the ascorbic acid and control arms regarding the mean change in hemoglobin level. On the other hand, the rate of blood transfusion was substantially reduced in favor of the ascorbic acid arm compared with the control arm. Nonetheless, this result should be carefully interpreted as the timing (i.e., intraoperative versus postoperative) and reason (i.e., surgical versus nonsurgical) of blood transfusion was not clearly stated by the meta-analyzed studies. Although the mean operative time was significantly reduced in favor of the ascorbic acid arm compared with the control arm, the difference does not seem to be clinically important. Potential theories for the reduced operative time could be related to the relative decrease in intraoperative bleeding and the better hemostatic surgical field, which collectively could have contributed to the faster completion of the surgery.

Ascorbic acid is a water-soluble vitamin; hence, it is largely safe and not stored in the body. An accumulating body of evidence reports that daily ingestion of 2 g of ascorbic acid is safe and free of serious adverse events [22,23]. In the present meta-analysis, two RCTs qualitatively reported that no serious adverse events related to ascorbic acid were identified [10,11]. One RCT [9] reported that the rate of fever was higher in the disfavor of the ascorbic acid arm compared with the control arm; however, the adverse event was neither serious nor statistically significant.

Overall, the present meta-analysis does not seem to recommend the use of ascorbic acid as an antihemorrhagic agent among patients undergoing myomectomy. Evidence from previous meta-analyses showed the clinical efficacy of several antihemorrhagic agents, such as oxytocin/carbetocin [24], tranexamic acid [25], and vasopressin [6], during myomectomy. Hence, future investigations may include a direct comparison between ascorbic acid and other active comparators (e.g., oxytocin/carbetocin, tranexamic acid, and vasopressin).

Strengths and Limitations

The present research has several strengths. This was the first meta-analysis that investigated the impact of ascorbic acid versus passive control on blood loss and related morbidities among patients undergoing myomectomy. The inclusion of only RCTs was advantageous in generating high-quality evidence. Three out of the four meta-analyzed outcomes were homogeneous. With the exception of intraoperative blood loss, all endpoints were commonly reported by the three RCTs.

Nonetheless, there are several shortcomings that ought to be acknowledged. Most notably, the small number of eligible RCTs and the corresponding sample sizes represent the major limitations. Additional limitations comprise the dissimilarities in clinical (e.g., different ascorbic acid doses) and surgical (e.g., different routes of myomectomy) characteristics that could have potentially influenced the degree of between-study heterogeneity and the summary conclusions. Another significant heterogeneity that needs to be acknowledged is the placebo-controlled design of two studies versus standard intervention in one study, which could have seriously jeopardized blinding and introduced bias. Lastly, publication bias was not tested because of the small number of included studies [26].

## Conclusions

This systematic review and meta-analysis of three RCTs revealed that ascorbic acid administration was linked to significant reductions in mean operative time and rate of blood transfusion compared with the control arm. Nonetheless, there was no significant difference between both arms regarding the mean intraoperative blood loss and mean change in hemoglobin level. In view of the limitations of the present meta-analysis, the use of ascorbic acid as an antihemorrhagic additive among patients undergoing myomectomy is not strongly recommended. Additional placebo-controlled trials with large sample sizes are needed to consolidate the quality of the evidence on the role of ascorbic acid in mitigating blood loss and related morbidities during myomectomy. Potential research may examine dose-response analysis to identify the dose that correlates with maximum antihemorrhagic effects and minimum toxicity.
